# Meta-Analysis of the Effects of Insect Pathogens: Implications for Plant Reproduction

**DOI:** 10.3390/pathogens12020347

**Published:** 2023-02-18

**Authors:** Wilnelia Recart, Rover Bernhard, Isabella Ng, Katherine Garcia, Arietta E. Fleming-Davies

**Affiliations:** 1 Biology Department, University of San Diego, 5998 Alcala Park, San Diego, CA 92110, USA; 2 Biology Department, Lewis and Clark College, 615 S. Palatine Hill Road, Portland, OR 97219, USA; 3 Environmental Sciences Department, University of California at San Diego, 9500 Gilman Drive, La Jolla, CA 92093-0021, USA

**Keywords:** arthropods, herbivores, herbivory, indirect effect, pathogens, pollination, pollinators, sublethal effects

## Abstract

Despite extensive work on both insect disease and plant reproduction, there is little research on the intersection of the two. Insect-infecting pathogens could disrupt the pollination process by affecting pollinator population density or traits. Pathogens may also infect insect herbivores and change herbivory, potentially altering resource allocation to plant reproduction. We conducted a meta-analysis to (1) summarize the literature on the effects of pathogens on insect pollinators and herbivores and (2) quantify the extent to which pathogens affect insect traits, with potential repercussions for plant reproduction. We found 39 articles that fit our criteria for inclusion, extracting 218 measures of insect traits for 21 different insect species exposed to 25 different pathogens. We detected a negative effect of pathogen exposure on insect traits, which varied by host function: pathogens had a significant negative effect on insects that were herbivores or carried multiple functions but not on insects that solely functioned as pollinators. Particular pathogen types were heavily studied in certain insect orders, with 7 of 11 viral pathogen studies conducted in Lepidoptera and 5 of 9 fungal pathogen studies conducted in Hymenoptera. Our results suggest that most studies have focused on a small set of host–pathogen pairs. To understand the implications for plant reproduction, future work is needed to directly measure the effects of pathogens on pollinator effectiveness.

## 1. Introduction

Pathogens are ubiquitous and infect many insects [1]. Empirical and theoretical studies of insect–pathogen interactions have informed our understanding of disease population dynamics [2,3], the use of pathogens for biological control [4,5], and the coevolution of hosts and pathogens [6,7,8]. Much of this prior work has focused on the pairwise interactions between pathogens and their insect hosts, overlooking the many ways in which these pathogens mediate how insects interact with other species, particularly plants (but see [9,10]).

Insects interact with plants when they consume plant tissue as herbivores and when they provide mutualistic services such as pollination and seed dispersal. These interactions can directly influence the distribution, survival, and reproduction of the interacting plant species [11,12,13]. Plant–pollinator interactions are among the most widely studied species interactions [14], and animal pollinators are crucial in the reproductive success of 87.5% of flowering plant species [15]. Moreover, insect herbivores can negatively affect plant reproduction through tissue damage or by disrupting plant–pollinator interactions, thus affecting plant fitness [16]. While a great deal of work has established the strong effects of pathogens on their insect hosts [17,18,19,20], few studies have examined the impacts of these pathogens on their hosts’ functions as pollinators and herbivores. Even fewer studies have directly measured the cascading effects on plant reproduction [21,22,23]. 

**Figure 1:**
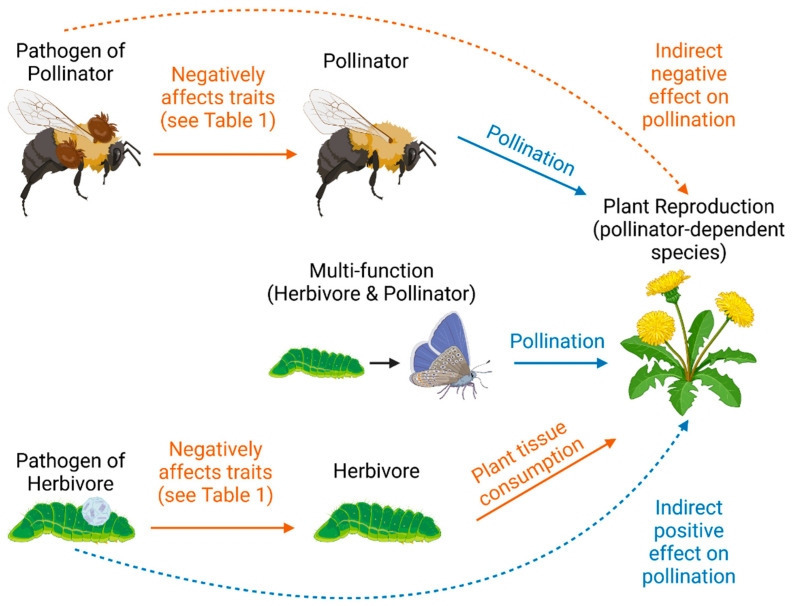
Conceptual diagram describing hypothetical ecological relationships between insect pathogens, insects, and plant reproduction, visualizing the tritrophic relationship pathways that could dictate how insect pathogens affect pollination. Solid lines indicate direct effects; dashed lines indicate hypothesized indirect effects. Orange lines indicate hypothesized negative effects, and blue lines indicate hypothesized positive effects.

Prior literature has largely used a tritrophic framework to study insect–pathogen interactions, examining how plant traits and defenses indirectly affect the insect–pathogen interactions [10,24,25] rather than how the plants are affected. Pathogens can influence plant–pollinator and plant–herbivore interactions through both density (i.e., quantitative) and trait-based (i.e., qualitative) effects (Figure 1). Most research on the effects of insect pathogens on plants has focused on density-based or quantitative indirect effects. Pathogens have been found to drastically influence insect population densities, both in field studies measuring extreme population cycling due to disease [3,26], as well as in numerous lab studies documenting pathogen effects on insect survival [17,27] and reproduction [28,29,30]. The negative effects of pathogens on insect population densities lead to clear predictions for plant reproduction because most species interactions and their outcomes are density-dependent: increases in pollinators typically lead to increases in flower visitation [31], and increases in herbivore densities lead to increases in defoliation [32,33]. However, these changes do not always produce corresponding effects on plant reproduction. If higher densities of herbivorous insects lead to increased defoliation, plants might be forced to allocate more resources towards herbivore defense, leaving fewer resources available for reproduction [34,35]. Yet despite this mechanistic prediction, these effects are inconsistent across all plant species and may even occur in the opposite direction through overcompensation responses to herbivory [36]. For example, a meta-analysis showed that herbivory can have either a positive or negative effect on floral traits depending on the type and location of damage caused to the plant [16]. In pathogens of pollinators, effects on insect survival and reproductive traits could similarly lead to density-based effects on plant reproduction. For example, *Apis mellifera* inoculated with *Nosema ceranae* survived less than noninoculated bees [27]. These reductions in insect density could lead to changes in the frequency of visits to flowers and thus decreases in pollinator services [37]. These effects are particularly relevant in pollinator-dependent plant species. 

Pathogen-induced changes in the abundance of a particular pollinator species could influence pollinator competitive environments and thus allow other pollinator species to increase their visitation to the plant if foraging decisions in the community are influenced by the presence of other pollinators. For example, the introduction of *Anthidium* bees decreased patch residence and the number of floral visits by *Megachile* bees to *Lotus corniculatus* [38]. If densities of *Anthidium* fluctuate with pathogen exposure, then we should expect *Megachile* to increase visitation to *L*. *corniculatus*. Additionally, a pathogen-driven decrease in the abundance of a pollinator could alter plant–pollinator networks and plant reproduction, similar to how adding (or losing) a pollinator species in a community can influence other species interactions [39,40]. Thus, pathogen effects on insect densities could influence community structure. 

Compared to density-based effects, there has been limited investigation of how pathogen-induced qualitative changes in insect traits might affect plants despite a vast literature on these trait-based effects, often termed ‘sublethal effects’ in lethal pathogens such as baculoviruses [41,42]. In pollinators, pathogens have been found to influence insect behavior [22,43] and morphology [44] in ways that have strong potential to influence pollination services. For example, proboscis length varies greatly in bees and it influences the types of flowers bees can access both across and within species [45,46]. Pollinator body shape, including body and wing size, can influence pollen placement on the body of the pollinator and pollen transfer between plants [45,47,48,49]. Insect physiology can dictate foraging decisions that could directly impact pollination [50]. Additionally, changes to wing morphology could influence the length of time or distance a pollinator can forage, which could influence gene flow and levels of inbreeding and outbreeding depression in plants [51]. These trait-based changes can have important repercussions for pollinator services. However, their effects on plant reproduction depend on whether the novel trait or change in trait value promotes or deters pollination. For example, changes in the behavior of *Eucera fervens* bees caused by exposure to *Nosema ceranae* can alter pollen movement between *Cucurbita maxima* plants [52]. Moreover, an observational study showed that higher prevalence of *Nosema* in *Bombus* spp. individuals led to decreases in the proportion seed set (total seed set by total number of open florets) of *Trifolium pratense*, a bumblebee-dependent plant. This occurred even though bumblebee visitation was consistent across years, suggesting a trait-based effect of *Nosema* on *Trifolium* pollination [21].

The unique lifecycles of insects mean that a single species can have both positive and negative effects on plants at different stages of development, which must be considered when predicting how insect pathogens affect plant reproduction. Insects that undergo complete metamorphosis have drastic changes in the ways they interact with plants [53]. For example, most Lepidoptera are herbivorous larvae that transform into nectivorous adults, many of which provide pollination services to plants [54]. Thus, in these cases, the effects of insect pathogens on plant fitness depend on whether the insect interacts with the same plant species as both an herbivore and a pollinator or with different plant species at different stages. In the first scenario, plant reproduction can be negatively affected by the Lepidopteran pathogen if the negative effects of the pathogen on pollen movement by the pollinator outweigh the positive effects of reduced herbivory. For example, *Datura wrightii* is pollinated by the hawkmoth *Manduca sexta*, which also lays its eggs on these plants, where the hatched larvae feed on leaf tissue [55]. Thus, a pathogen-induced reduction in the densities of the hawkmoths will influence both pollination and herbivory by these insects. The effects of insects that function as both herbivores and pollinators should also depend on pollinator community composition. For example, if the plant has other pollinator species available, then the negative effect of pathogens on the insect species could lead to a net increase in plant reproduction. Thus, herbivory would decrease and pollinator services could potentially be sustained by other pollinator species. In the second scenario, if the insect interacts with different plant species as an herbivore and as a pollinator, a single insect pathogen could have positive impacts on the reproduction of one plant species and negative impacts on another depending on which plant species experience herbivory or pollination by the insect. For example, *Dione vanillae* is a Nymphalid Lepidoptera with an herbivorous larval stage that consumes *Passiflora* spp. leaf tissue [56]. The adult butterflies of this species pollinate other plants such as species of *Asclepias*, *Epidendrum*, and *Lantana* [57,58]. Thus, a pathogen of *Dione vanillae* might have positive effects on *Passiflora* fitness but negative effects on *Asclepias*. In our analysis, we describe insect species with both an herbivore and a pollinator stage as ‘multi-function’ species and consider this functional group as separate from herbivore or pollinator species (Figure 1).

Here we present a meta-analysis of how pathogen exposure influences the traits of insect pollinators and herbivores, in order to determine the potential impacts of insect pathogens on plant reproduction. Specifically, we addressed the following questions: (1) what is the effect of pathogens on the traits of insect pollinators and herbivores? (2) Does the effect of pathogens vary by pathogen type, insect order, or trait measured? Lastly, (3) does the functional group of insects (pollinator, herbivore, or multi-function) influence the effects of pathogens on insects? We then identify knowledge gaps and discuss future research directions to connect plant reproduction and insect–pathogen interactions. 

## 2. Methods

### 2.1. Literature Search and Data Collection

This review was performed in accordance to the PRISMA (Preferred Reporting Items for Systematic Reviews and Meta-Analyses) guidelines. We used the Web of Science database to search for relevant bodies of literature using the following search criteria: 

Topic [TS] = (lepidoptera* OR bees OR bee OR fly OR butterfl* OR moth OR hymenopter* OR Bombus OR Apis OR bumble* OR aphid* OR caterpillar* OR larva* OR arthropod* OR insect* OR pollinati* OR herbivor* OR orthoptera OR spider* OR hemiptera OR coleoptera OR beetle) AND TS = (trait* OR morpholo* OR proboscis OR foraging OR learn* OR abundance OR sub-lethal OR sublethal) AND Author Keywords [AK] = (disease* OR viru* OR pathogen* OR parasite* OR infect*) AND Title [TI] = (disease* OR viru* OR pathogen* OR parasite* OR infect*).

These search criteria yielded 1879 hits from scientific literature published from 1997 to 9 July 2020. A second search was conducted to obtain articles previous to 1997, which yielded 1181 papers (range 1989–1996; see Figure S1 for PRISMA chart). Scientific literature considered was published in English and included articles, book chapters, reviews, published conference proceedings or abstracts, dissertations, and theses. From this search, we employed the following criteria of inclusion:

(1) Insect species must belong to an insect order with known species that are herbivores or pollinators.
(2) The article contained quantitative data on the effects of pathogens, specifically sample size, mean, and some measure of variability (e.g., standard deviation or standard error) on insect behavior, demography, physiology, or morphology (see Table 1). This data needed to be collected for both uninfected and infected insects. Infected insects could be exposed to any dose of the pathogen. Observational studies were included if they measured traits of both naturally infected and uninfected insects.
(3) Only pathogens for which insects are the primary host were considered. Plant or vertebrate diseases for which insects act as the disease vector were excluded.
(4) If multiple pathogen species were studied, data must have been collected on each pathogen species separately. Coinfection data were excluded.

The following data were extracted from each research article: (1) insect taxonomy, (2) pathogen taxonomy, (3) host function, (4) infected life stage, (5) effect type and description, (6) life stage of the observed effect, (7) experiment type (observational or manipulative) and location (field or lab), (8) pathogen treatment and dose, and (9) quantitative data from which to calculate effect size (sample size, mean, and standard deviation or standard error). Extracted data (1) through (8) were used to document how different variables could play a role in the sublethal effects of pathogens on the traits of insect pollinators or herbivores. Detailed descriptions for each of the variables collected can be found in Table 1.

**Table 1:** Description of all variables collected from each research article that was included in the meta-analysis.

Variable Name	Description	Additional Details or Examples
(1) insect taxonomy	Taxonomy of the infected insect, including insect order and scientific species name.	e.g., Hymenoptera, *Bombus sonorus*
(2) pathogen taxonomy	Taxonomy of the pathogen, including pathogen group and scientific species name.	Groups: bacteria, fungus, virus, multicellular parasite (e.g., nematodes, trematodes, and mites)
(3) host function	The functional group of the insect: pollinator, herbivore, or multi-function.	Multi-function insects are insects that act as herbivores or pollinators depending on their life stage. For example, many Lepidoptera can be herbivores as larvae and pollinators as adults.
(4) infected stage	The life stage at which the infection/transmission occurs in the insect: larva, pupa, or adult.	We group infection/transmission at the egg stage with larval stage.
(5) trait category and description	Effect of the pathogen on the insect. The pathogen could have demographic, physiological, morphological, or behavioral effects.	Examples for each trait category: Demographic: fecundity and mortality Physiological: growth or metabolic rate Morphological: body size, proportions, or deformities Behavioral: foraging rate or flight endurance
(6) insect stage data were collected	The life stage the trait was measured in.	Larva, pupa, or adult
(7) study type and location	The type of study and the location where the study was conducted.	Type of study: manipulative or observationalStudy location: field or laboratory
(8) pathogen treatment	Pathogen treatment was used as a categorical variable and based on the doses of the pathogen treatment present in the study.	Pathogen treatment: uninfected or infected. For experiments with multiple doses, the highest dose was used.

### 2.2. Statistical Analyses

#### 2.2.1. Overview and Meta-Analytic Model

We calculated the effect size for each comparison between infected and uninfected insects (i.e., case study or k) as Hedges’ *g*, which accounts for variation within samples and in sample sizes between and across case studies [59]. In our analyses, effects were measured such that positive values were associated with higher insect fitness and negative values were associated with decreases in insect fitness. For example, if a study measured the proportion of insects dying with and without pathogen exposure, we converted this to the proportion of insects surviving so that higher values are associated with higher fitness. We then used these effect sizes to fit a meta-analytic linear mixed-effects model that considered the random effects of case study, research article, and case study nested within research article as sources of variation. This model was fit using the rma.mv function in the metafor package [59]. After exploring potential sources of bias in our dataset (see details below), we used the same mixed-effect model structure to consider whether the effects of insect pathogen on insects were influenced by (1) insect taxonomy, (2) pathogen taxonomy, (3) insect functional group, (4) infected life stage, (5) effect type (demographic, morphological, behavioral, or physiological), (6) life stage at which the effect was measured, (7) study type, and (8) setting of the study (see Table 1). 

#### 2.2.2. Exploring Bias in Our Dataset

To detect and understand bias in our dataset, we implemented two approaches. First, we calculated Rosenberg’s fail-safe number, also called a file drawer analysis. This fail-safe number provides a value that is compared to a threshold value calculated using an equation that incorporates the case study value (5 × k + 10). If the fail-safe number is bigger than the threshold value, then we can conclude that while bias might be present in our analysis, it should not be strong enough to influence the interpretation of our results (Rosenberg 2005). Our fail-safe number (N_observed_ = 44,420) was bigger than our threshold value (N_threshold_ = 1110).

We also removed outliers from the dataset and fitted the meta-analytic models (as described above) to see the role that outliers played in the interpretation of our results. We did this by converting each effect size into a z-score and identified any z-scores > |3.29| as outliers in our dataset (see Figure S2). This method identified two outliers. The removal of these outliers did not have a strong impact on the interpretation of our main fixed effects, although the interpretation of some of the categories within these fixed effects changed. Specifically, without the outliers, we did not detect a significant negative effect of pathogens on the insect order of Coleoptera (Hedges’ *g* = −0.51, 95%CI: −1.44, 0.43), and we detected a significant effect size for pollinator host function (Hedges’ *g* = −0.38, 95%CI: −0.71, −0.05) and the insect order Orthoptera (Hedges’ *g* = –1.14, 95%CI: −2.07, −0.21).

## 3. Results

### 3.1. Literature Survey

**Figure 2:**
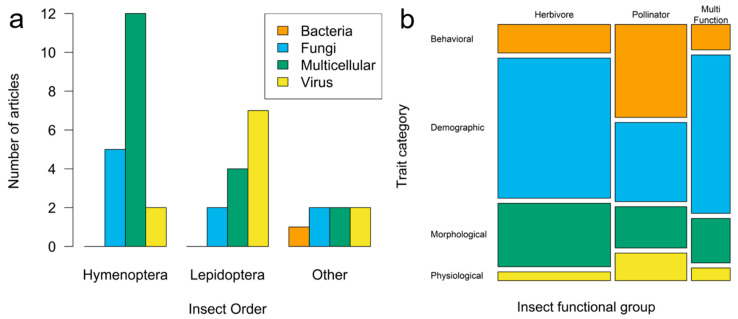
(**a**) Frequency bar plots of article counts by insect and pathogen taxonomy. “Other” combines Coleoptera, Orthoptera, Blattodea, and Hemiptera. (**b**) Mosaic plot of the number of trait comparisons (k) by insect functional group and trait category.

Our search yielded 218 comparisons between infected and uninfected insects (k) from 39 research articles (n). These articles represented studies spanning 21 insect species representing 6 insect orders as well as 25 pathogen species, including viruses, bacteria, fungi, and multicellular parasites (Figure 2 and Table 2). 

Herbivores were the most common functional group represented (n = 15 articles, k = 110 comparisons), with most of these being Lepidoptera (n = 8, k = 78), followed by pollinators (n = 19, k = 70; only Hymenoptera) and, lastly, those serving as both pollinators and herbivores (n = 4, k = 33). The most common studies were manipulative (n = 34, k = 196), were conducted in the lab (n = 35, k = 175), exposed insects to pathogens during their adult stage (n = 23, k = 88), measured the effects of the pathogen on adult insects (n = 34, k = 149), and documented demographic effects (n = 25, k = 112).

Articles were fairly evenly distributed across pathogen types except for bacteria, which were underrepresented compared to all other pathogen types (chi-square goodness-of-fit test; X^2^ = 15.05, df = 3, *p* <0.01; Figure 2a). However, particular pathogen types tended to be more heavily studied in certain insect orders, with 81 of 94 case studies (7 of 11 articles) of viral pathogen effects conducted in Lepidoptera (primarily herbivores) and 15 of 29 case studies (5 of 9 articles) of fungal pathogens conducted in Hymenoptera (all pollinators; Figure 2a; Fisher’s exact test on article numbers, *p* = 0.03). In addition, studies of pollinators (all Hymenoptera) appeared more likely to measure behavioral traits (k = 27 of 70 traits measured in pollinators) compared to studies of herbivores (k = 13 of 110; Figure 2b; no statistics conducted as comparisons k are nested in articles and thus nonindependent).

### 3.2. Effects of Pathogen Identity and Insect Order and Function 

**Figure 3:**
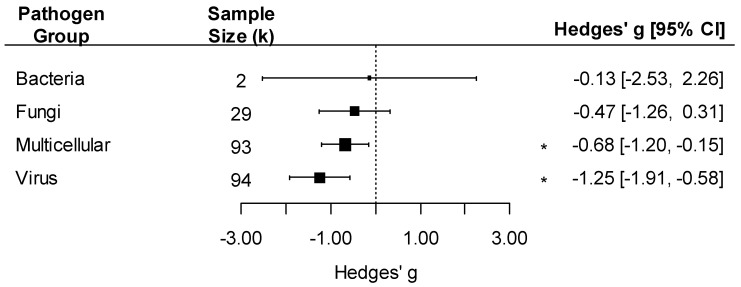
Forest plot showing pathogen effect sizes on insect traits by pathogen group (bacteria, fungi, multicellular, or virus). Positive values correspond to positive effects on fitness. Asterisks * represent significant effects at *p* < 0.05, and the vertical dashed line highlights a zero Hedges’ *g* value (no difference between infected and uninfected groups). Larger squares indicate a larger sample size (number of comparisons, k).

**Figure 4:**
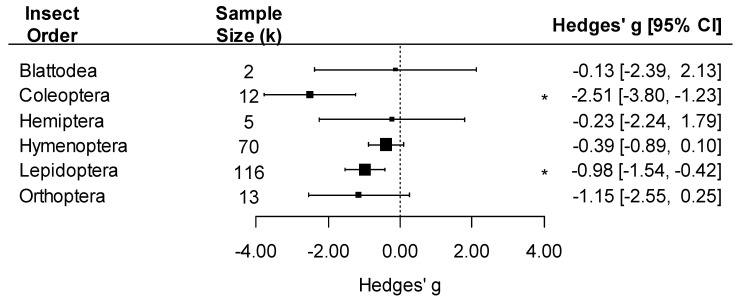
Forest plot showing pathogen effect sizes on insect traits by insect order. Positive values correspond to positive effects on fitness. Asterisks * represent significant effects at *p* < 0.05, and the vertical dashed line highlights a zero Hedges’ *g* value (no difference between infected and uninfected groups). Larger squares indicate a larger sample size (number of comparisons, k).

We detected an overall negative effect of pathogen exposure on insect traits (k = 218, Hedges’ *g* = −0.79, 95%CI: −1.15, −0.43; *p* < 0.001). The effect of pathogens varied by pathogen group, with a negative effect detected only on insects infected by viruses (Hedges’ *g* = −1.25) or multicellular parasites (excluding fungi, Hedges’ *g* = −0.68 (Figure 3; pathogen group effect: F_4, 214_ = 5.37, *p* < 0.001)). Since bacteria had very few comparisons (k = 2), we did not interpret the effect size for this group. The effect of pathogens on insect traits was also influenced by the taxonomy of the insect, with significant negative effects of pathogens on Coleoptera and Lepidoptera but not in other insect orders (Figure 4; insect order effect: F_6, 212_ = 5.33, *p* < 0.001). While Blattodea is reported in this analysis, due to its small k value (k = 2), an interpretation of its effect size was not made.

**Figure 5:**
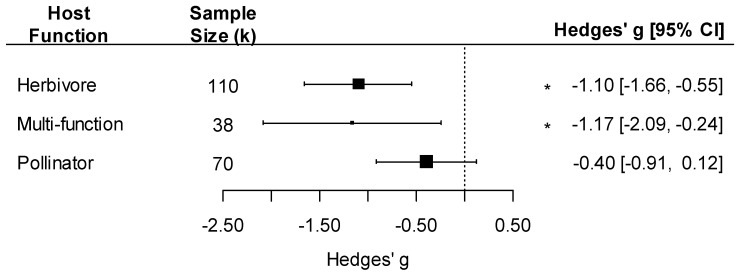
Forest plot showing pathogen effect sizes on insect traits by host function (herbivore, multi-function, or pollinator). Positive values correspond to positive effects on fitness. Asterisks * represent significant effects at *p* < 0.05, and the vertical dashed line highlights a zero Hedges’ *g* value (no difference between infected and uninfected groups). Larger squares indicate a larger sample size (number of comparisons, k).

Moreover, the effect of pathogens on insect traits varied by host functional group: we detected a negative effect of pathogens only on insects that were herbivores or carried multiple functions in the community (as both herbivores and pollinators at different life stages) and not on insect pollinators (Figure 5; host function effect: F_3, 215_ = 7.94, *p* < 0.001). 

Intriguingly, the difference in effects with pathogen and insect taxonomy and function all seem to be driven by the same group of studies with weak or no effects of pathogens: infections of pollinators (all Hymenoptera) with fungal pathogens (15 of 70 comparisons; k = 15, Hedges’ *g* = −0.12, 95%CI: −0.31, 0.08; *p* > 0.05). When fungal pathogens were removed, a negative effect size was detected in pollinators (k = 55, Hedges’ *g* = −0.43, 95%CI: −0.70, −0.16; *p* = 0.002).

### 3.3. Effects by Trait Type and Life Stage of Observed Traits and Pathogen Exposure

**Figure 6:**
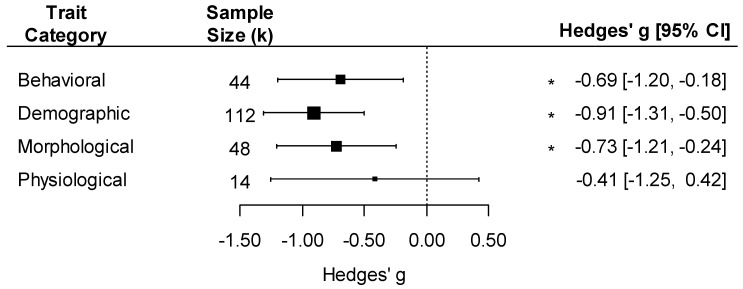
Forest plot showing pathogen effect sizes on insect traits for different trait categories (behavioral, demographic, morphological, and physiological). Positive values correspond to positive effects on fitness. Asterisks * represent significant effects at *p* < 0.05, and the vertical dashed line highlights a zero Hedges’ *g* value (no difference between infected and uninfected groups). Larger squares indicate a larger sample size (number of comparisons, k).

The effect of pathogens on fitness varied by the type of effect measured, with a negative effect of pathogens on demographic, morphological, and behavioral traits but not on physiological traits (Figure 6; trait type effect: F_4, 214_ = 5.44, *p* < 0.001). The effect size was also influenced by the life stage in which the trait was measured, with a negative effect present in all life stages (Figure S3; F_3, 215_ = 6.47, *p* < 0.001). Moreover, the negative effect of pathogens on insect traits varied by the life stage in which they were exposed to the pathogen. We detected a significant and similar negative effect on insects infected as adults or larvae but not on those infected as pupae, which were relatively rare in the dataset (Figure S4; life stage exposed to pathogen effect: F_3, 215_ = 6.02, *p* < 0.001; k_pupae_ = 6). 

### 3.4. Effects by Study Type and Setting 

We detected negative effects of pathogens on insect traits in manipulative but not in observational studies (Figure S5; study type effect: F_2, 216_ = 9.15, *p* < 0.001). While both study types had a similar mean effect, observational studies had larger confidence intervals, likely due to the much smaller sample size (k = 23 for observational versus k = 195 for manipulative). Moreover, the effect of pathogens on insect traits was influenced by the setting of the study, with negative effects detected in laboratory studies but not in field studies (Figure S6; setting of the study effect: F_2, 216_ = 9.09, *p* < 0.001). A lack of effect detected from field studies is likely due to the much smaller sample size (k = 43 for field versus k = 175 for lab studies). Field studies are also likely to have higher variation as it is not possible to control conditions as tightly as in laboratory studies, possibly leading to higher standard error.

## 4. Discussion

The strong negative effects of pathogens on insect traits we detected have important implications for plant reproduction. Out of our four trait types, we detected negative effects of pathogens on insect demographic traits, morphology, and behavior but not on physiology. Our failure to detect negative effects on physiological traits might be due to lower statistical power in that category (k = 14 case studies), suggesting a need for additional studies in this area. In general, the effects of pathogens on insect demographic traits were greatly overrepresented compared to the other effect types, contributing more than half of all comparisons (Figure 6). These demographic traits such as survival and fecundity directly influence insect population densities. Thus, their overrepresentation is consistent with the relatively larger literature on quantitative indirect effects of pathogens compared to qualitative indirect effects. To fully understand the implications for plant reproduction, more research is needed to quantify the impacts of pathogens on pollinator behavior, morphology, and physiology and directly measure how these impacts connect to plant reproduction. Ideally, this work would start with determining behavioral, morphological, and physiological traits known to influence pollination services. Examples of these traits could include proboscis length, wing length, and pollinator foraging decisions such as constancy and time spent at each flower. Currently, these connections have been established through observational studies looking at natural variation in pathogen load [21,52] as well as theoretical work focusing on behavior in Hymenoptera [22]. Thus, we suggest that researchers conduct direct manipulations of pollinator exposure to pathogens to measure the impacts of pathogens on these pollination-relevant traits as well as the corresponding cascading effects on plant reproduction. This approach has been used to understand the effects that bee parasitoids have on plant reproduction [92].

In our meta-analysis, we detected stronger negative effects in lab studies than in field studies. An underrepresentation of field studies was caused by our data-gathering approach where studies that did not include a control group (no exposure to the pathogen) could not be included, leading to a decrease in statistical power for this group. Additionally, greater negative effects in lab studies could have been detected if these experiments administer high doses of the pathogen compared to what insects might encounter in the field. This was further exacerbated by selecting the highest dose for the meta-analysis, and laboratory studies were more likely to have experimental designs with multiple doses. Interestingly, the life stage in which insects were exposed to the pathogen did not influence the strength of the effect despite prior research typically showing stronger pathogen effects at juvenile stages (e.g., [93,94]). 

### 4.1. Taxonomic Gaps in Current Insect–Pathogen Literature

While pathogens infect all insect orders, our meta-analysis found that most insect–pathogen studies have focused on just a few species, leaving many insect and pathogen species understudied. For example, while Hymenoptera is a highly diverse and pollination-relevant insect order, the literature has primarily focused on species with commercial relevance such as *Apis mellifera* and bumblebees (e.g., *Bombus impatiens* and *Bombus terrestris*) [75,81,86,88]—with *Bombus* studies establishing lab colonies from field-collected individuals. Studies on noncommercial species have often focused on natural populations of *Bombus* or on leaf-cutting bees (Megachilidae) [74,81,91]. Research on this insect order has been conducted primarily in lab settings (see Table 2). Thus, to fully understand the impacts of pathogens on plant pollination, it is crucial to determine the impact of pathogens on native and nonagricultural Hymenoptera species, ideally using targeted field manipulations. Viruses of Hymenoptera were also underrepresented in our analysis (Figure 2a), which is of concern given the strong ecological and conservation impacts of pathogens such as deformed wing virus [95]. 

A similar research trend was detected within the Lepidoptera, an order that contains many species of herbivores and ‘multi-function’ species with a larval herbivore stage and an adult pollinator stage. Most research on insect–pathogen interactions within this order (78 of 116 identified case studies) has focused on a few herbivorous nonpollinating moth species such as the western tent caterpillar *Malacosoma californicum* (k = 28) and the tobacco cutworm *Spodoptera litura* (k = 22), with the extensive work on the monarch butterfly *Danaus plexippus* being the notable exception (k = 38). Moreover, most of the relevant studies of these herbivorous Lepidoptera have focused on species-specific baculoviruses (81 of 116 identified case studies), lethal viruses that can lead to massive outbreaks in the exposed Lepidopteran larvae [96]. Although most of these outbreaking forest insects are not pollinators, other members in the Lepidoptera are of pollination significance [97,98], most or all of which have their own host-specific nucleopolyhedrosis virus (NPV) [96]. Thus, the extensive ‘sublethal effects’ (i.e., quantitative and qualitative effects of pathogen exposure on surviving individuals) of NPV exposure documented in the literature likely apply to Lepidopteran pollinators. More work is needed to measure these effects in pollinating species and to directly connect these effects to plant reproduction or pollination services of the Lepidopteran flower visitors. For example, activity levels in female monarchs (including nectar feeding) decrease with infection by the protozoan parasite *Ophryocystis elektroscirrha* (OE disease), although this decreased activity has not yet been connected to effects on pollination [17].

In addition to the unbalanced representation of host species within orders, many common herbivore-containing insect orders (e.g., Hemiptera) [99]^,^ and insect-pollinating orders (e.g., Diptera) [100] were underrepresented or entirely absent in our analysis. Much of the herbivore–pathogen research was limited to agriculturally relevant or model organisms that are easy to grow and maintain in laboratory settings despite the high diversity of insect herbivores [99]. In our analysis, we found strong effects of viruses and multicellular parasites on insect fitness but did not detect fungi effects. This was likely a result of the abundance of data on certain pathogen types rather than a true difference in the strength of effects. Some examples of these overrepresented pathogens include OE disease, a multicellular parasite in monarch butterflies (4 studies and 28 comparisons), *Crithidia bombi*, a multicellular parasite in bees (10 studies and 44 comparisons), and baculoviruses in other Lepidoptera species (7 studies and 81 comparisons). While the choice of study systems is likely driven at least in part by true biological differences in the prevalence of particular pathogens between insect orders, in most cases, this justification is omitted. Thus, future research should provide a broader description of all parasites known to interact with the species of study. Furthermore, there are clear gaps in our knowledge of common host–pathogen systems, such as microsporidia in Lepidoptera. Thus, to better understand the implications of insect pathogens for plant reproduction, we recommend future studies on an expanded range of insect and pathogen species, particularly in novel combinations. 

Intriguingly, the effects of pathogens appear to be weaker in the most common type of pollinator–pathogen study in our analysis: a pollinating Hymenoptera species (e.g., *Apis*) infected with a fungal pathogen (e.g., *Nosema*). The weaker effects in this well-studied combination appear to be driving the lack of detectable effects within comparisons among insect orders (no effect in Hymenoptera), pathogen types (no effect in fungal pathogens), and insect function (no effect in pollinator-only species). This does not appear to be purely due to a lack of statistical power, as the confidence intervals were similar in size to the other groups in which significant effects were detected. This result appears to contradict much of the established literature in which *Nosema apis* and *N. ceranae* have been associated with colony collapse disorder and other highly virulent outcomes in *Apis mellifera* [101]. In many of these studies, *Nosema* spp. were found in association with other pathogens, and thus the fact that our meta-analysis excluded coinfection experiments might be driving this weaker effect [102,103]. If the effects of pathogens truly are weaker for this particular host–pathogen combination, this requires further investigation to determine if this pattern is true across Hymenoptera species, for example, or if it is mainly driven by this particular host–pathogen combination. 

### 4.2. Predicting Effects of Herbivore and Pollinator Pathogens on Pollination

While we found strong negative effects of pathogens across a range of insect traits, predicting how these effects might influence pollination and plant reproduction is far from straightforward. In nature, these pairwise plant–pollinator and plant–herbivore interactions exist in a web of other species interactions. Thus, linear changes in the density or traits of a particular insect species might result in complex and nonlinear effects on plant reproduction and fitness. 

Moreover, evolutionary processes might enhance or counteract the ecological effects of pathogen-driven changes in pollinators or herbivores. For example, changes in pollinator abundance or composition could lead to changes in pollinator-mediated selection on plants [104,105,106,107]. If pathogen exposure is consistent enough, selection on insects for disease resistance could indirectly result in changes that influence their effectiveness as pollinators due to correlated traits. These changes could generate novel traits or an increase in phenotypic variation in the insect populations. For example, laboratory infections and quantitative genetics work have found that in some Lepidoptera species, lower susceptibility to pathogens is correlated with smaller body size, lower growth rates, and lower fecundity [8,108,109]. If larger size is associated with higher pollinator effectiveness, as it is in many generalist bees and flies [110], selection for disease resistance could thus have negative effects on plant reproduction. In contrast, in a highly coevolved specialist plant-pollinator interaction, changes in pollinator size, in either direction, could negatively impact pollination [111]. Thus, disease-induced morphological changes, whether through the direct effects of a pathogen or indirectly through selection on resistance, can have neutral, positive, or negative implications for plant reproduction depending on how that trait influences pollination in a particular system. These changes should be studied within the context of eco-evolutionary dynamics between the plant, the pollinator, and the pathogen.

Climate change adds further complications to predicting the impacts of insect pathogens on plant reproduction. Increased temperatures can lead to increased insect pathogen outbreaks [112,113]. Thus, under climate change, pathogens have an even greater potential for influencing plants that interact with these insects. Plant reproduction and plant growth can also be influenced directly via changes to the environment [114,115,116,117], which can, in turn, mediate host–pathogen interactions of plant-interacting insects [9,10]. Thus, current and future climatic change offer a perplexing puzzle in which multiple species interactions will need to be considered to fully understand their consequences on the ecology and evolution of species. 

## 5. Future Directions and Recommendations 

Based on the results of the meta-analysis, we make the following recommendations for future research on how insect pathogens affect plant reproduction.

### 5.1. Targeted Studies of Pathogen–Insect–Plant Interactions 

Although there is strong evidence of insect pathogens influencing traits with the potential to affect pollination services, there is still little direct evidence for the impact of insect pathogens on pollination. Thus, we suggest that researchers conduct direct manipulations of pollinator exposure to pathogens in order to measure the impacts of pathogens on pollination-relevant traits as well as the corresponding cascading effects on plant reproduction. A similar approach can be used to study the impacts of pathogen exposure on herbivores.

### 5.2. Greater Communication between Disease and Pollination Ecologists 

Many studies of pathogen effects on morphology, behavior, and population density focus on insect species that are either pollinators or are related to pollinating species (e.g., Lepidoptera). Increased communication between these fields would enhance our understanding of the ecological context and ramifications of pathogens. 

### 5.3. Expanded Combinations of Insect–Pathogen Pairs

Unsurprisingly, there is a need for increased breadth of study systems, particularly for native and nonagricultural Hymenoptera. In addition, even for well-studied host insects and pathogens, most research tends to fall into particular host–pathogen combinations (e.g., NPVs and OE disease in Lepidoptera; *Nosema* in Hymenoptera). If researchers of well-studied insect host systems considered additional pathogens alongside the well-studied combinations, this would provide information allowing us to separate host versus pathogen species effects, which could then be connected to plant reproduction.

### 5.4. Increased Use of Manipulative Field Experiments

More research should focus on field or natural settings to determine whether the stronger pathogen effects from lab studies reflect field or natural insect–pathogen dynamics and interactions. Although logistically difficult, there is a particular need for field studies that manipulate pathogen exposure.

### 5.5. Consider Environmental and Evolutionary Context

Climate change continues to challenge our current understanding of species interactions. It is important to understand how interconnected species interactions, such as those described in this meta-analysis, are affected by environmental change. The potential for rapid evolution in both insects and pathogens also makes it essential to consider evolutionary processes in determining the ecological effects of these interactions.

In sum, while there is strong potential for insect pathogens to influence pollination services and plant reproduction, there is a great need for further work to directly study these connections. This will also aid in the broader goal of connecting species interactions to ecological communities.

## Figures and Tables

**Table 2:** Summary table describing the study system and number of comparisons for each research article included in the meta-analysis.

Insect Functional Group	Insect Order	Insect Species Name	Pathogen Name	Number of Comparisons by Trait Category (k)				Type of Study	Location of Study	Reference
				Behav.	Demogr.	Morphol.	Physiol.			
Herbivore	Blattodea	*Zootermopsis angusticollis*	*Serratia marcescens*	0	2	0	0	Manipul.	Lab	Cole et al. 2018 [60]
	Coleoptera	*Agriotes obscurus*	*Metarhizium brunneum*	0	1	0	0	Manipul.	Lab	Zurowski et al. 2020 [61]
		*Phyllophaga vandinei*	Iridovirus 6	1	2	0	0	Manipul.	Lab	Jenkins et al. 2011 [62]
		*Tribolium castaneum*	*Steinernema feltiae*	0	0	8	0	Manipul.	Lab	Kramarz et al. 2014 [63]
	Hemiptera	*Myzus persicae*	MpDNV	4	0	1	0	Manipul.	Lab	Dupont et al. 2020 [64]
	Lepidoptera	*Anticarsia gemmatalis*	AgNPV	0	8	4	0	Manipul.	Lab	Peng et al. 1997 [65]
		*Helicoverpa armigera*	HearNPV	0	3	0	0	Manipul.	Lab	Eroglu et al. 2018 [66]
		*Lymantria dispar*	LdNPV	0	0	2	0	Manipul.	Lab	Paez et al. 2015 [67]
		*Lymantria dispar*	*Nosema* sp.	0	1	0	4	Manipul.	Lab	Goertz et al. 2004 [68]
		*Malacosoma californicum*	McplNPV	6	10	12	0	Manipul.	Field	Rothman 1997 [28]
		*Orgyia antiqua*	*Metarhizium anisopliae*	0	2	0	0	Manipul.	Lab	Sandre et al. 2011 [69]
		*Spodoptera litura*	SlGV	0	20	2	0	Manipul.	Lab	Gupta et al. 2010 [29]
		*Thaumetopoea pityocampa*	TpCPV	2	2	0	0	Manipul.	Lab	Ince et al. 2007 [70]
	Orthoptera	*Camnula pellucida*	*Entomophaga grylli*	0	6	0	0	Manipul.	Field	Kistner et al. 2014 [71]
		*Nemobius sylvestris*	*Paragordius tricuspidatus*	0	7	0	0	Observat.	Field	Biron et al. 2005 [72]
Multi-function	Lepidoptera	*Danaus plexippus*	*Ophryocystis elektroscirrha*	0	6	6	2	Manipul.	Lab	Altizer 2001 [44]
		*Danaus plexippus*	*Ophryocystis elektroscirrha*	0	4	1	0	Manipul.	Lab	Altizer and Oberhauser 1999 [17]
		*Danaus plexippus*	*Ophryocystis elektroscirrha*	3	0	0	0	Manipul.	Lab	Bradley et al. 2005 [18]
		*Danaus plexippus*	*Ophryocystis elektroscirrha*	0	6	0	0	Manipul.	Lab	Tao et al. 2015 [73]
		*Pieris brassicae*	PbGV	1	9	0	0	Manipul.	Lab	Sood et al. 2010 [30]
Pollinator	Hymenoptera	*Andrena scotica*	Microsporidia	0	0	2	0	Observat.	Field	Paxton et al. 1997 [74]
		*Apis mellifera*	Deformed wing virus	0	2	2	0	Manipul.	Lab	Tehel et al. 2019 [75]
		*Apis mellifera*	*Nosema apis*	4	0	0	0	Manipul.	Lab	Wang et al. 1970 [76]
		*Apis mellifera*	*Nosema ceranae*	3	3	0	0	Manipul.	Lab	Ferguson et al. 2018 [27]
		*Apis mellifera*	*Tropilaelaps mercedesae*	0	0	2	0	Manipul.	Lab	Khongphinitbunjong et al. 2016 [77]
		*Apis mellifera*	*Varroa destructor*	3	0	0	0	Manipul.	Lab	Kralj 2006 [78]
		*Apis* sp.	Deformed wing virus	1	0	0	0	Manipul.	Lab	Coulon et al. 2020 [79]
		*Apis* sp.	*Nosema ceranae*	1	0	0	0	Manipul.	Lab	Naug 2014 [80]
		*Bombus impatiens*	*Crithidia bombi*	1	0	0	0	Manipul.	Lab	Figueroa et al. 2019 [81]
		*Bombus impatiens*	*Crithidia bombi*	4	0	0	0	Observat.	Lab	Gegear et al. 2005 [82]
		*Bombus impatiens*	*Crithidia bombi*	3	0	0	0	Both	Lab	Gegear et al. 2006 [83]
		*Bombus impatiens*	*Crithidia bombi*	0	2	0	0	Manipul.	Lab	Giacomini et al. 2018 [84]
		*Bombus impatiens*	*Crithidia bombi* & *Locustacarus buchneri*	6	0	0	0	Observat.	Lab	Otterstatter et al. 2005 [85]
		*Bombus* spp.	*Crithidia bombi*	1	1	0	0	Observat.	Lab	Shykoff et al. 1991 [86]
		*Bombus terrestris*	*Crithidia bombi*	0	2	2	0	Manipul.	Lab	Brown et al. 2000 [87]
		*Bombus terrestris*	*Crithidia bombi*	0	6	1	0	Manipul.	Lab	Brown et al. 2003a [88]
		*Bombus terrestris*	*Crithidia bombi*	0	3	3	9	Manipul.	Lab	Brown et al. 2003b [89]
		*Bombus terrestris*	*Crithidia bombi*	0	3	0	0	Manipul.	Lab	Yourth et al. 2008 [90]
		*Osmia bicornis*	*Nosema ceranae*	0	2	0	0	Manipul.	Lab	Mueller et al. 2019 [91]

## Data Availability

The data presented in this study are openly available in DataDryad, at https://doi.org/10.5061/dryad.9s4mw6mmw.

## Supplementary Materials

The following supporting information can be downloaded at: https://www.mdpi.com/article/10.3390/pathogens12020347/s1, Figure S1: PRISMA flow chart for meta-analysis [118]; Figure S2: Histogram showing the z-values (a) using all case studies and (b) after the removal of the two identified outliers; Figure S3: Forest plot showing pathogen effect sizes by the life stage in which the trait was measured; Figure S4: Forest plot showing pathogen effect sizes by the infected life stage; Figure S5: Forest plot showing pathogen effect sizes by study type; Figure S6: Forest plot showing pathogen effect sizes by study setting.
