# Supplementary material for: Meta-Analysis of the Effects of Insect Pathogens: Implications for Plant Reproduction
Source: Pathogens. 2023 Feb 18;12(2):347. doi: 10.3390/pathogens12020347 (PMC9958737; doi:10.3390/pathogens12020347)
Supplement: Supplementary file 1 [file pathogens-12-00347-s001.zip › pathogens-2178084-supplementary.pdf]

Supplementary materials

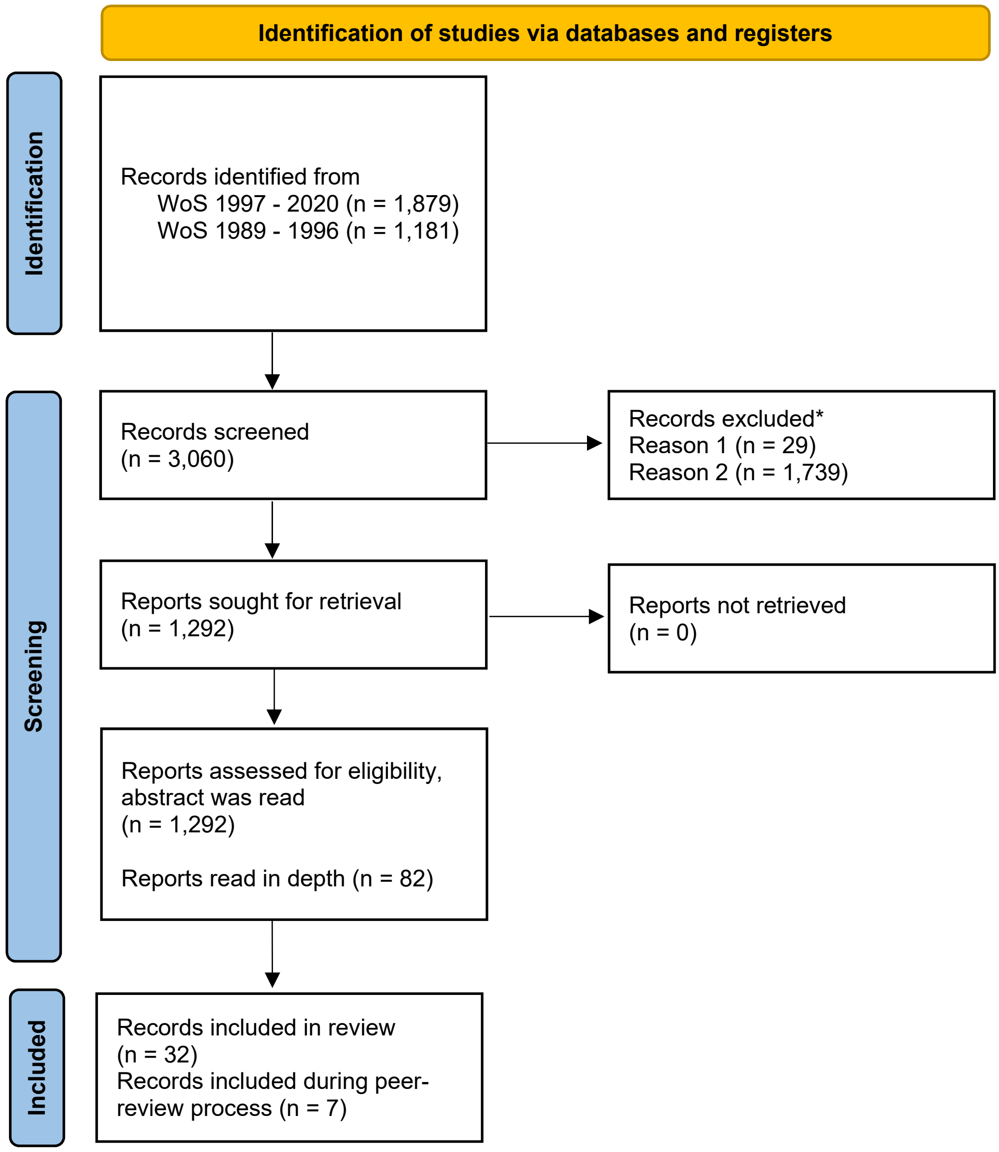

*Exclusion reasons:
Reason 1, duplicated entries
Reason 2, the organism is not an insect or the pathogen does not infect an insect, determined from reading the article title

Figure S1. PRISMA flow chart for meta-analysis. Adapted from [118].

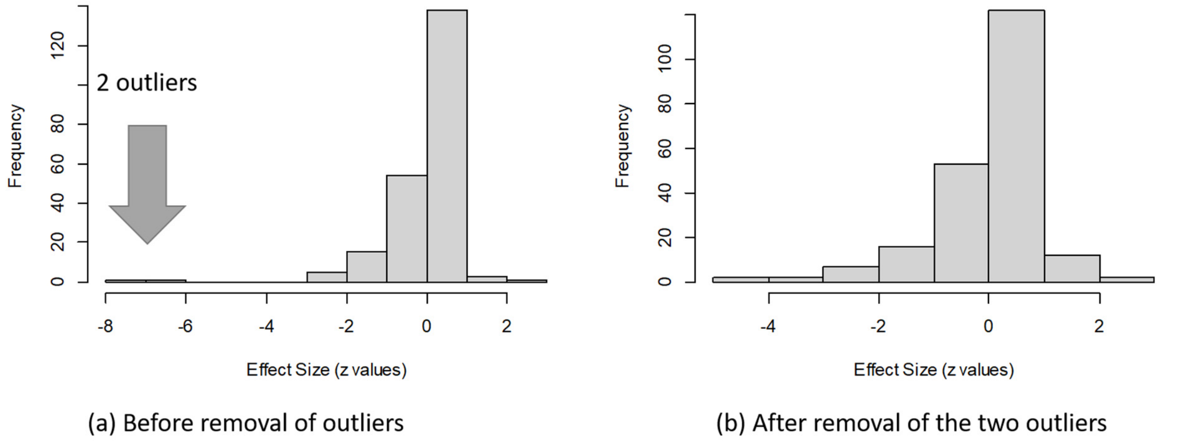

Figure S2. Histogram showing the z-values (a) using all case studies and (b) after the removal of the two identified outliers.

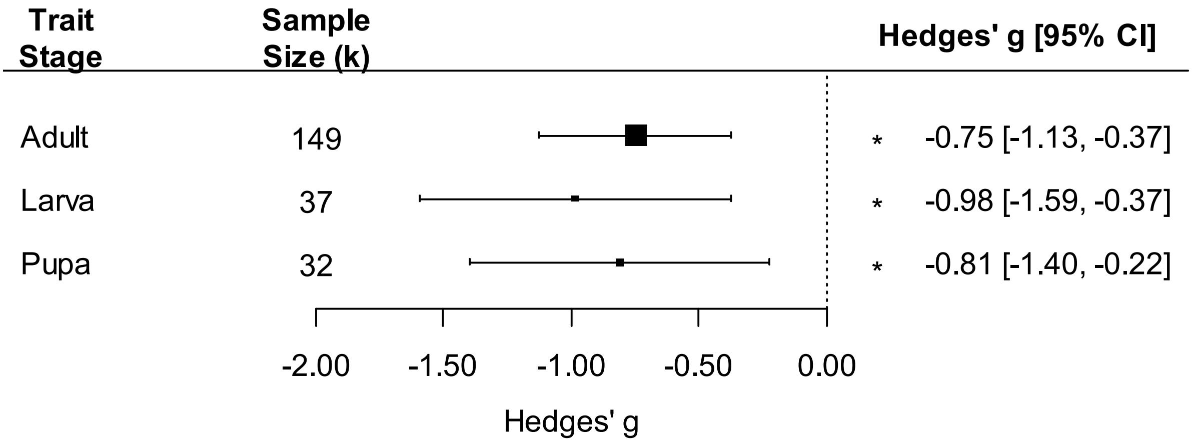

Figure S3. Forest plot showing pathogen effect sizes by the life stage in which the trait was measured (adult, larva, pupa). Asterisks represent significant effects at $p < 0.05$.

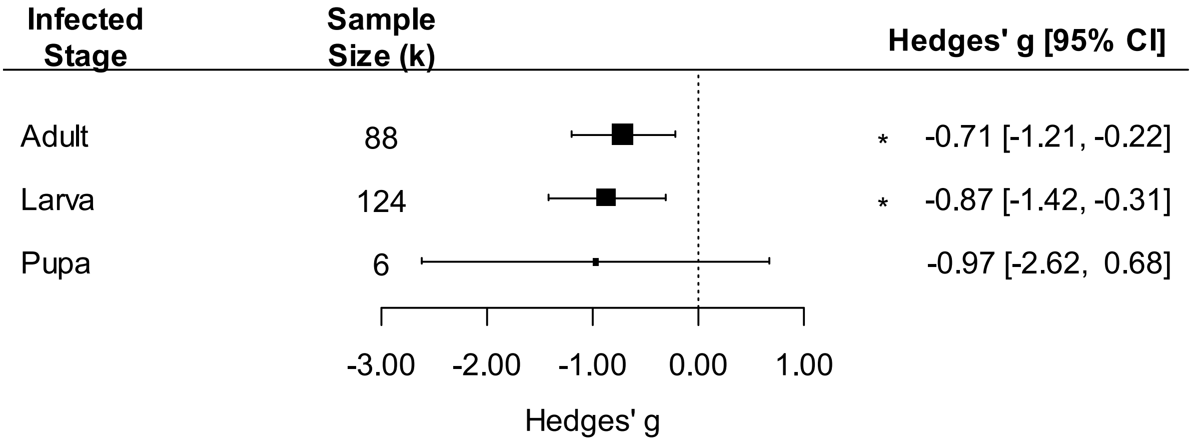

Figure S4. Forest plot showing pathogen effect sizes by the infected life stage (adult, larva, pupa). Asterisks represent significant effects at $p < 0.05$.

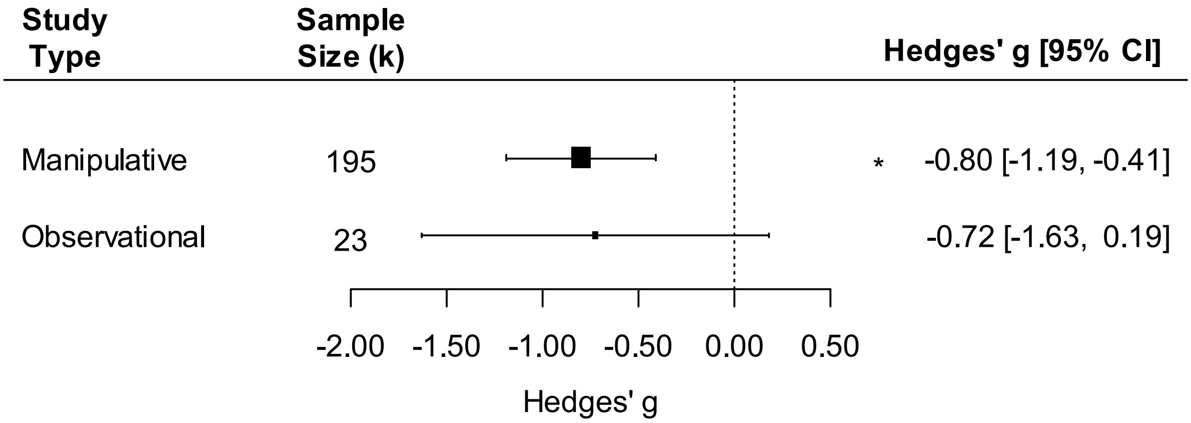

Figure S5. Forest plot showing pathogen effect sizes by study type. Asterisks represent significant effects at $p < 0.05$.

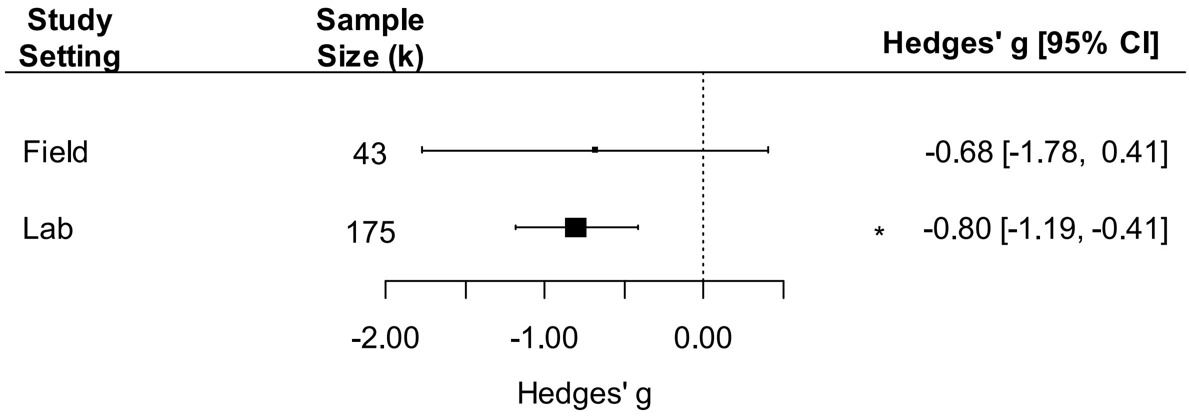

Figure S6. Forest plot showing pathogen effect sizes by study setting (laboratory or field). Asterisks represent significant effects at $p < 0.05$.

Reference

118 Page, M.J.; McKenzie, J.E.; Bossuyt, P.M.; Boutron, I.; Hoffmann, T.C.; Mulrow, C.D. et al. The PRISMA 2020 statement: an updated guideline for reporting systematic reviews. *BMJ* **2021**, *72*, n71. doi: 10.1136/bmj.n71
